# Gene expression patterns of red sea urchins (*Mesocentrotus franciscanus*) exposed to different combinations of temperature and *p*CO_2_ during early development

**DOI:** 10.1186/s12864-020-07327-x

**Published:** 2021-01-07

**Authors:** Juliet M. Wong, Gretchen E. Hofmann

**Affiliations:** 1 Department of Ecology, Evolution and Marine Biology, University of California Santa Barbara, Santa Barbara, CA 93106 USA; 2 Present address: Department of Biological Sciences, Florida International University, North Miami, FL 33181 USA

**Keywords:** Red sea urchin, *Mesocentrotus franciscanus*, RNA-seq, Transcriptomics, Early development, Climate change, Warming, Ocean acidification

## Abstract

**Background:**

The red sea urchin *Mesocentrotus franciscanus* is an ecologically important kelp forest herbivore and an economically valuable wild fishery species. To examine how *M. franciscanus* responds to its environment on a molecular level, differences in gene expression patterns were observed in embryos raised under combinations of two temperatures (13 °C or 17 °C) and two *p*CO_2_ levels (475 μatm or 1050 μatm). These combinations mimic various present-day conditions measured during and between upwelling events in the highly dynamic California Current System with the exception of the 17 °C and 1050 μatm combination, which does not currently occur. However, as ocean warming and acidification continues, warmer temperatures and higher *p*CO_2_ conditions are expected to increase in frequency and to occur simultaneously. The transcriptomic responses of the embryos were assessed at two developmental stages (gastrula and prism) in light of previously described plasticity in body size and thermotolerance under these temperature and *p*CO_2_ treatments.

**Results:**

Although transcriptomic patterns primarily varied by developmental stage, there were pronounced differences in gene expression as a result of the treatment conditions. Temperature and *p*CO_2_ treatments led to the differential expression of genes related to the cellular stress response, transmembrane transport, metabolic processes, and the regulation of gene expression. At each developmental stage, temperature contributed significantly to the observed variance in gene expression, which was also correlated to the phenotypic attributes of the embryos. On the other hand, the transcriptomic response to *p*CO_2_ was relatively muted, particularly at the prism stage.

**Conclusions:**

*M. franciscanus* exhibited transcriptomic plasticity under different temperatures, indicating their capacity for a molecular-level response that may facilitate red sea urchins facing ocean warming as climate change continues. In contrast, the lack of a robust transcriptomic response, in combination with observations of decreased body size, under elevated *p*CO_2_ levels suggest that this species may be negatively affected by ocean acidification. High present-day *p*CO_2_ conditions that occur due to coastal upwelling may already be influencing populations of *M. franciscanus*.

**Supplementary Information:**

The online version contains supplementary material available at 10.1186/s12864-020-07327-x.

## Background

The red sea urchin *Mesocentrotus franciscanus* (A. Agassiz, 1863) is an ecologically and economically valuable species found along the Pacific Coast of western North America [1]. In subtidal areas, especially within kelp forests, these echinoderms are herbivorous ecosystem engineers that can shape the flow of resources within marine habitats [2]. Overgrazing by *M. franciscanus*, often in combination with overgrazing by the purple sea urchin *Strongylocentrotus purpuratus*, can lead to the formation of urchin barrens in which macroalgal communities are severely reduced or depleted [3, 4]. Red sea urchins also function as prey to animals at higher trophic levels, including spiny lobsters and sea otters [5–7]. In addition to its removal by natural predators, *M. franciscanus* is widely collected as a lucrative wild fishery species. Fisheries in Mexico, the United States, and Canada harvest *M. franciscanus* for their gonads (i.e., roe) that supply domestic markets as well as international exports, principally to Japan [8, 9]. Over recent years (2015–2019), the annual revenue reported from *M. franciscanus* fisheries across the states of California, Oregon, and Washington averaged over $7.1 million USD/year, far more than all other echinoderm fishery species combined [10].

Given the considerable ecological and economic importance of *M. franciscanus*, determining how this species will be affected by continuing environmental change in coastal oceans remains an overlooked and critical area of research [11]. Due to their habitat and life history, these urchins are threatened by climate change impacts [12] such as ocean warming, which may include sudden and extreme marine heat waves [13, 14], and ocean acidification, which may amplify the low pH conditions that episodically occur in upwelling regions [15]. The upwelling season in the California Current System (CCS) typically extends from early spring until late summer or fall; it is characterized by fluctuations between periods of upwelling, when cold, low pH water is transported to the surface, and periods in which upwelling is relaxed (i.e., wind conditions are not conducive for driving upwelling) [16, 17]. This overlaps with the natural spawning period of *M. franciscanus* that occurs annually during spring and early summer months [18–20]. Therefore, in the CCS, *M. franciscanus* embryos and larvae experience combinations of temperature and pH conditions that may vary depending on whether spawning and upwelling events coincide. Furthermore, these urchins may be particularly vulnerable to stress during early development. Although planktonic embryological and larval stages of echinoids are capable of exhibiting vertical migration [21, 22], they are likely less capable of finding refuge from stressful conditions than their benthic adult counterparts. There is also evidence that many organisms are most vulnerable to environmental stress early in their life history [23–26]. Both lethal and sublethal effects that occur during early development or that carry over into later life stages will negatively affect the recruitment necessary to support future populations [27, 28].

Given the dynamic nature of their habitat and the progression of ocean warming and acidification, it is imperative to understand how early stage *M. franciscanus* respond to their environment on a molecular level. A limited number of studies have investigated how *M. franciscanus* responds to temperature or *p*CO_2_ stress [29–31], and even fewer have done so within a multi-stressor context [32]. In *S. purpuratus*, a species whose habitat largely overlaps with that of *M. franciscanus*, elevated temperatures and *p*CO_2_ levels during early development can cause increased mortality, abnormality, and a reduction in size and scope for growth [20, 33–35]. Several studies have identified and examined temperature- and *p*CO_2_-responsive genes in *S. purpuratus* embryos and larvae [36–41]. Studies such as these are essential for contributing molecular-level insights into how these organisms respond, or fail to respond, to stressful environmental conditions and may help explain effects observed at the level of the organism or population. This is particularly pertinent for fishery species in which accurate predictions are necessary for adaptive, climate-ready fisheries management [42]. Although a clear understanding of how *M. franciscanus* responds to environmental stress is lacking, suggestions have already been made to replace or offset the *M. franciscanus* fishery with *Strongylocentrotus fragilis*, a sea urchin species expected to be more tolerant to climate change [12]. Here, both temperature and *p*CO_2_ conditions were manipulated in a laboratory setting to investigate their influence on the gene expression patterns of *M. franciscanus* during its early development. To the best of our knowledge, this is the first study to use RNA sequencing (RNA-seq) to examine the *M. franciscanus* stress response.

In this study, *M. franciscanus* embryos were raised under a combination of two temperatures (13 °C or 17 °C) and two *p*CO_2_ levels (475 μatm or 1050 μatm) that reflect current and future ocean conditions in their natural habitat [15, 43–45]. This generated four different treatment combinations: 1) 17 °C and 1050 μatm *p*CO_2_, 2) 17 °C and 475 μatm *p*CO_2_, 3) 13 °C and 1050 μatm *p*CO_2_, and 4) 13 °C and 475 μatm *p*CO_2._ In the highly dynamic CCS, treatment combinations #2–4 are currently measured during and between upwelling events [15, 43–45]. Future ocean conditions are represented by treatment combination #1 (i.e., the simultaneous occurrence of 17 °C and 1050 μatm *p*CO_2_) given continued ocean warming and acidification. Additional detail regarding the selection of these temperature and *p*CO_2_ treatment levels is located in the Methods. Gene expression patterns were assessed at both the gastrula and prism embryo stages. We also discuss the gene expression results within the context of previously reported physiological assessments from this experiment, including body size and thermotolerance [46]. Here, we describe the effects of the temperature and *p*CO_2_ treatments at the molecular level and whether they relate to observations made at the level of the organism.

Temperature elicited a robust transcriptomic response at both developmental stages. Gene expression analyses indicated that the warmer temperature (i.e., 17 °C) induced a cellular stress response, amongst other processes. Additionally, the variation in gene expression that was significantly correlated to the temperature treatment was also significantly correlated to embryo body size and thermotolerance, characteristics that were neutrally or positively influenced by the warmer temperature treatment [46]. In contrast, the transcriptomic response to the *p*CO_2_ treatment was comparatively muted. This minor molecular-level response may explain the reduction in embryo body size that is observed under elevated *p*CO_2_ levels (i.e., 1050 μatm) [46]. Overall, we examined a valuable fishery species that is capable of dramatically shaping coastal ecosystems, and determined that during early development *M. franciscanus* exhibits different magnitudes of transcriptomic plasticity in response to two climate change-related stressors. This study provides much needed insight into a species that is important for many fisheries on the Pacific coast of North America, facilitating our understanding of how *M. franciscanus* development is affected by current ocean conditions, as well as our predictive capacity of how this species will respond to future ocean change.

## Results

### Summary statistics and overview of RNA-seq

The samples used for RNA-seq were generated from triplicate cultures of embryos raised at each of the four combined temperature and *p*CO_2_ treatments (i.e., 12 total cultures) (see Additional file 1). Each sample was collected as a pool of 5000 embryos from each of the 12 cultures at both the gastrula and prism stages during development to produce a total of 24 samples used for RNA extractions and library preparation. Sequencing of the 24 libraries yielded a total of 728,782,735,100-bp single reads. After quality trimming, an average of 30.3 ± 1.3 million reads per library remained. FASTQC reports [47] of trimmed sequences showed high sequence quality (> 30) with limited adapter contamination or presence of overrepresented sequences. Per-library mapping efficiency to the developmental transcriptome [48] using RSEM [49] was at an average of 52.6%. The presence of mitochondrial rRNA appeared to have contributed to the percentage of unmapped reads, although mapping rate may have also been affected by the completeness of the reference transcriptome.

### Developmental stage influenced transcriptomic patterns

**Fig. 1:**
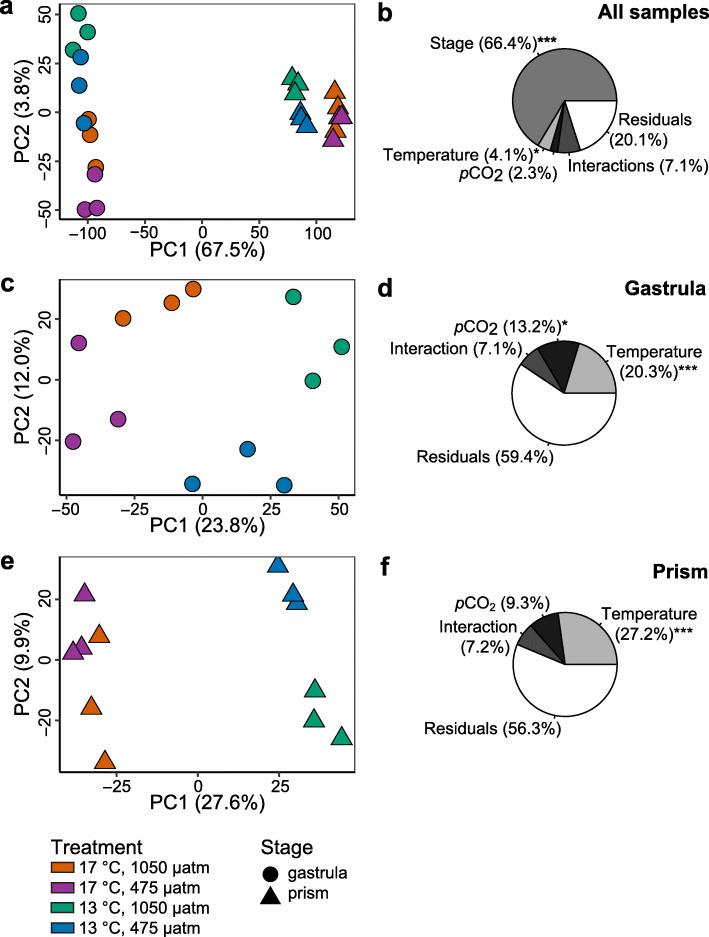
General gene expression patterns. Principal component analysis (PCA) plots of **a** all samples, **c** the gastrula stage only, and **e** the prism stage only are displayed with the two components that explained the most variance. Pie charts (**b**, **d**, and **f**) display the percent of variation explained by fixed factors determined using permutational multivariate ANOVAs (*p < 0.05 and ***p < 0.001). For **b** all samples, fixed factors included developmental stage, temperature treatment, and *p*CO_2_ treatment. The interactions of the three fixed factors have been consolidated into a single, “Interactions” pie chart segment for figure simplicity. For **d** the gastrula stage and **f** the prism stage, fixed factors only included temperature and *p*CO_2_ treatment

A principal component analysis (PCA) of sample-to-sample distances showed that differences in gene expression profiles were primarily between the two developmental stages, gastrula and prism (Fig. 1a). Principal Component (PC) 1 captured the majority of the variance (67.5%) and revealed a clear separation between gastrula and prism stage embryos, while PC2 only captured 3.8% of the variance. Indeed, a permutational multivariate ANOVA across all 24 samples with developmental stage, temperature treatment, and *p*CO_2_ treatment as fixed factors, revealed that developmental stage explained 66.4% of the variance (*p* = 0.001) (Fig. 1b). In contrast, temperature treatment explained only 4.1% of the variance (*p* = 0.041) and *p*CO_2_ treatment explained only 2.3% of the variance (*p* = 0.207). All factor interactions were not significant (*p* > 0.05). Results from gene expression analyses across all samples independent of stage (i.e., gastrula and prism stages were not analyzed separately) are available in Additional file 2. Because we have previously explored the differences in gene expression patterns across *M. franciscanus* during early development [48] and it is not the main focus of the current study, from here onward we report separate gene expression analyses for the gastrula and prism stages.

### Temperature and *p*CO_2_ affected gastrula gene expression

**Fig. 2:**
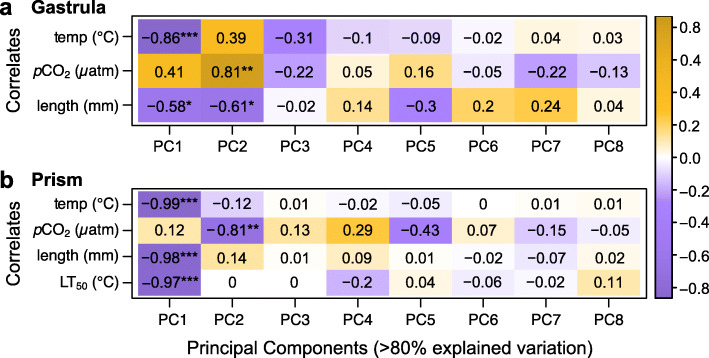
Correlations at **a** the gastrula stage and **b** the prism stage between PC1-PC8 (columns), which contribute > 80% of the explained variation in gene expression, and metadata variables (rows) of the experiment treatments (i.e., temperature and *p*CO_2_), body size (i.e. embryo length in mm), and thermotolerance (i.e. LT_50_ in °C, prism stage only). The orange-purple color scale represents the strength of the Pearson’s correlation (1 to − 1). *p < 0.05, **p < 0.01, and ***p < 0.001

Separate PCA plots were generated for the gastrula and prism stages. At the gastrula stage, we generally observed that both temperature and *p*CO_2_ treatments appeared to drive differences in gene expression patterns across samples. A PCA of only the gastrula stage showed that replicate samples grouped together (Fig. 1c). Here, PC1 captured 23.8% of the variance and was found to have a highly significant negative correlation to the temperature treatment (Fig. 2a). PC2 captured 12.0% of the variance (Fig. 1c) and was found to have a significant positive correlation with the *p*CO_2_ treatment (Fig. 2a). Using average embryo length measurements from this experiment (previously reported in [46]), both PC1 and PC2 were also found to be negatively correlated with gastrula body size (Fig. 2a).

Upon examining the PC loadings for the gastrula stage using the *PCAtools* package [50], the genes most responsible for variation along PC1 included an elongation factor 1-alpha gene and a transcription factor SUM-1-like gene (Additional file 3). Genes contributing variation to PC2 included a poly(A)-specific ribonuclease PARN gene and a putative DNA polymerase gene. A rank-based gene ontology (GO) analysis was performed following the *GO_MWU* package [51] in R. Using complete sets of loading values calculated from the PCA (Additional file 3), this analysis identified GO categories enriched by genes contributing variance to the PCs. GO terms related to regulation of gene expression, ion binding, and DNA recombination were enriched by variable genes in PC1 (Additional file 4a). Genes contributing variation to PC2 enriched GO categories associated with heat shock protein binding, peptide metabolic process, and amide biosynthetic process (Additional file 4b).

**Fig. 3:**
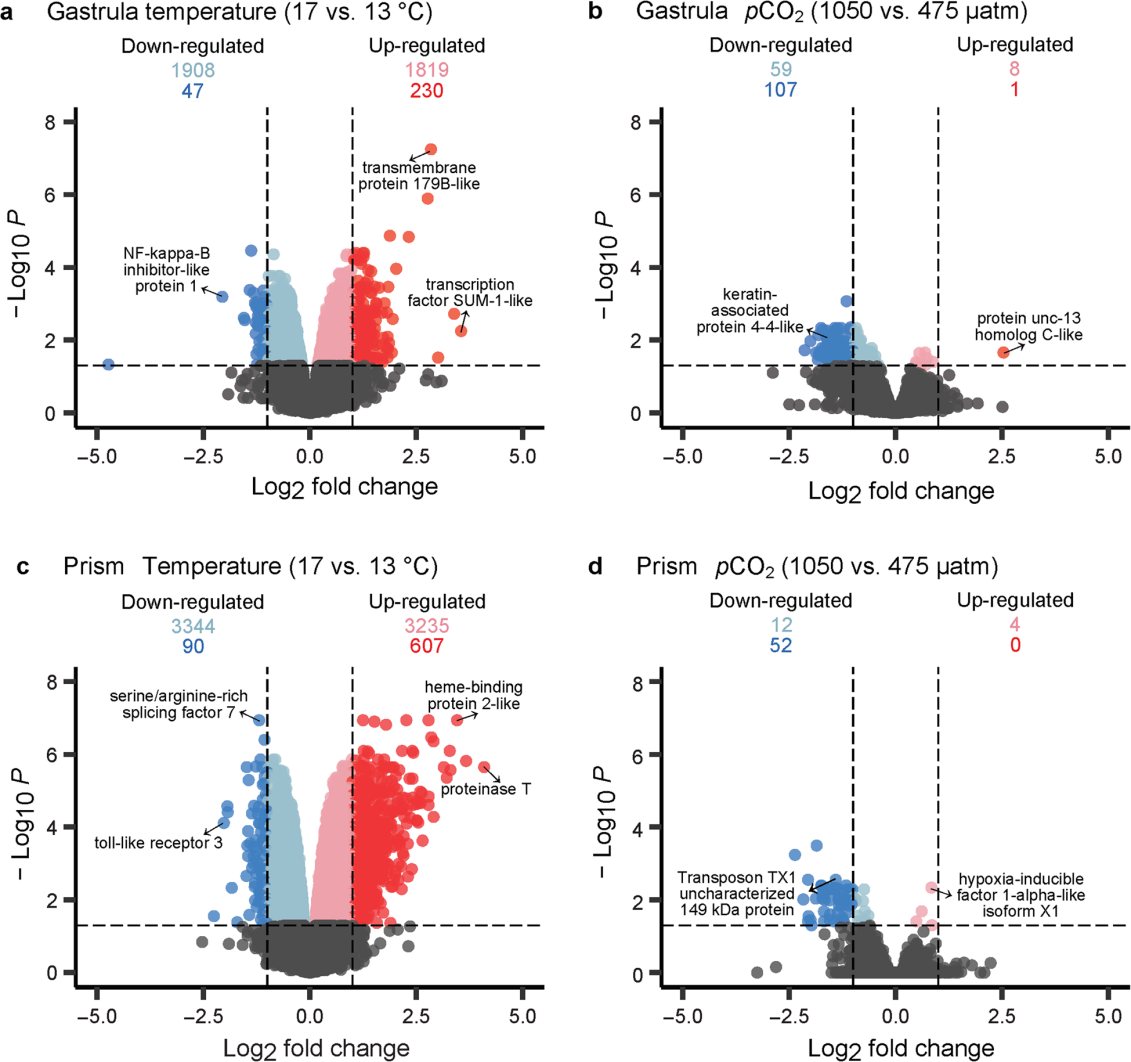
Temperature, and to a lesser degree, *p*CO_2_ treatments caused differential gene expression at **a**, **b** the gastrula stage and **c**, **d** the prism stage of early development. Genes that were not differentially expressed are displayed in gray while significant DE genes (adjusted *p*-value < 0.05) are displayed in color with a few selected genes labelled. Significant DE genes that were up-regulated are shown in pink (0 < log_2_ FC < 1) and red (log_2_ FC ≥ 1) and significant DE genes that were down-regulated are shown in light blue (− 1 < log_2_ FC < 0) and blue (log_2_ FC ≤ − 1)

A permutational multivariate ANOVA revealed that at the gastrula stage, 20.3% of the variance was explained by temperature treatment (*p* = 0.001) (Fig. 1d). Differential expression (DE) analyses conducted in *limma* [52] identified differentially expressed genes (relatively up- and down-regulated) between gastrula raised under different temperature treatments. A total of 2049 genes were significantly up-regulated in embryos raised at 17 °C relative to embryos raised at 13 °C (adjusted *p* < 0.05) (Fig. 3a). These up-regulated genes included a transcription factor SUM-1-like gene (log_2_ fold change (FC) = 3.39, adj. *p =* 0.002), a transmembrane protein 179B-like gene (log_2_ FC = 2.85, adj. *p* < 0.001), a cell death protein 3 gene (log_2_ FC = 1.58, adj. *p* = 0.031), and a heat shock 70 kDa protein 12A-like gene (log_2_ FC = 0.661, adj. *p* = 0.023) (Additional file 3).

**Fig. 4:**
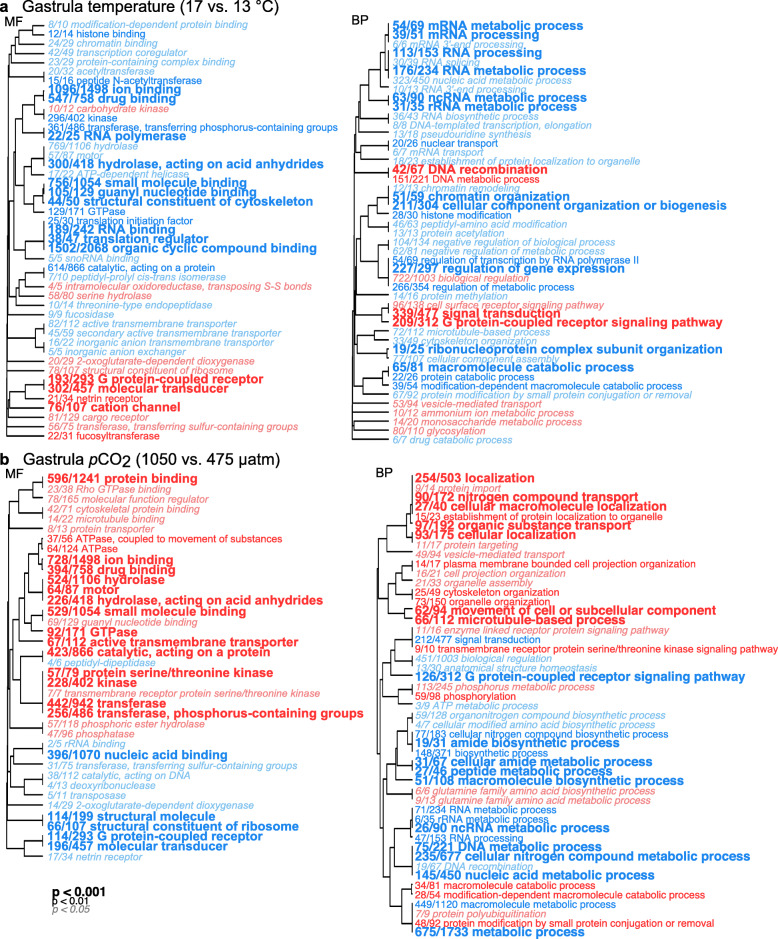
GO results of genes expressed at the gastrula stage. Analysis determined significant enrichment within GO categories of genes up-regulated (red text) and down-regulated (blue text) due to **a** temperature and **b** *p*CO_2_ treatments in gastrula embryos. Font sizes of the category names indicate the level of statistical significance as noted in the legend. The fraction preceding each category name is the number of genes with moderated t-statistic absolute values > 1 relative to the total number of genes belonging to the category. GO categories of molecular function (MF) and biological process (BP) are shown

Following DE analysis, gene ontology (GO) analyses were performed using the *GO_MWU* package [51] to identify GO categories that were enriched by up-regulated or down-regulated genes. Terms across molecular function (MF), biological process (BP), and cellular component (CC) GO categories were identified using moderated t-test values from the full list of genes (i.e., not exclusively DE genes with adjusted *p* < 0.05). GO categories significantly enriched with up-regulated genes influenced by temperature included DNA recombination, DNA metabolic process, cation channel, and G protein-coupled receptor signaling pathway (Fig. 4a, Additional file 5a).

A total of 1955 genes were down-regulated in embryos raised at 17 °C relative to embryos raised at 13 °C (Fig. 3a). These included a NF-kappa-B inhibitor-like protein 1 gene (log_2_ FC = − 2.06, adj. *p* < 0.001), a heat shock 70 kDa protein cognate 5 gene (log_2_ FC = − 0.27, adj. *p* = 0.019), and a heat shock 70 kDa protein 14 gene (log_2_ FC = − 0.40, adj. *p* = 0.003) (Additional file 3). GO categories enriched with down-regulated genes included regulation of gene expression, chromatin organization, histone modification, and ion binding (Fig. 4a, Additional file 5a).

The *p*CO_2_ treatment also affected gene expression patterns at the gastrula stage, explaining 13.2% of the observed variance (*p* = 0.021) (Fig. 1d). Only 9 genes were up-regulated when comparing the 1050 μatm to the 475 μatm *p*CO_2_ treatment at the gastrula stage, including a protein unc-13 homolog C-like gene (log_2_ FC = 2.54, adj. *p* = 0.022) (Fig. 3b, Additional file 3). GO analyses identified terms significantly enriched (*p* < 0.05) with genes affected by the *p*CO_2_ treatment. GO categories enriched with up-regulated genes included macromolecule catabolic process, ion binding, and active transmembrane transporter (Fig. 4b, Additional file 5b). A total of 166 genes were down-regulated in embryos raised at 1050 μatm relative to embryos raised at 475 μatm (Fig. 3b), including a keratin-associated protein 4–4-like gene (log_2_ FC = − 1.62, adj. *p* = 0.008) and a carbonic anhydrase 14-like isoform X3 gene (log_2_ FC = − 0.89, adj. *p* = 0.047) (Additional file 3). Enriched GO categories included macromolecule biosynthetic process, macromolecule metabolic process, and nucleic acid binding (Fig. 4b, Additional file 5b). The interaction between temperature and *p*CO_2_ factors explained 7.1% of the variance observed at the gastrula stage, but the interaction was not significant (*p* = 0.440) (Fig. 1d).

### Temperature was the primary factor affecting prism gene expression

Similar to the gastrula stage, the PCA of only the prism stage showed a separation of samples by treatment with sample replicates grouping together (Fig. 1e). PC1, which captured 27.6% of the variance, was found to have highly significant negative correlations to the temperature treatment, prism body size, and thermotolerance (Fig. 2b). Prism body size for each sample was estimated using average embryo length and LT_50_ (i.e., the temperature at which 50% mortality occurred) measurements from this experiment that were previously reported in [46]. PC2 captured 9.9% of the variance (Fig. 1e) and had a significant negative correlation with the *p*CO_2_ treatment (Fig. 2b).

Loadings within PC1 showed that genes responsible for most of the variation included a heme-binding protein 2-like gene and putative tolloid-like protein 1 genes (Additional file 3). Genes contributing variance to PC2 included an elongation factor 1-alpha gene, a F-box/WD repeat-containing protein 7-like gene, and a FK506-binding protein 5-like gene (Additional file 3). Using *GO_MWU* and the loading values for the complete set of genes, enriched GO categories for PC1 were identified. These included GO terms related to ion transport, regulation of gene expression, methylated histone binding, RNA methyltransferase, pseudouridine synthesis, antioxidant, cellular response to DNA damage stimulus, and response to oxidative stress (Additional file 4c). Genes contributing variation to PC2 enriched GO categories associated with ion binding, oxidoreductase, and metabolic process (Additional file 4d).

**Fig. 5:**
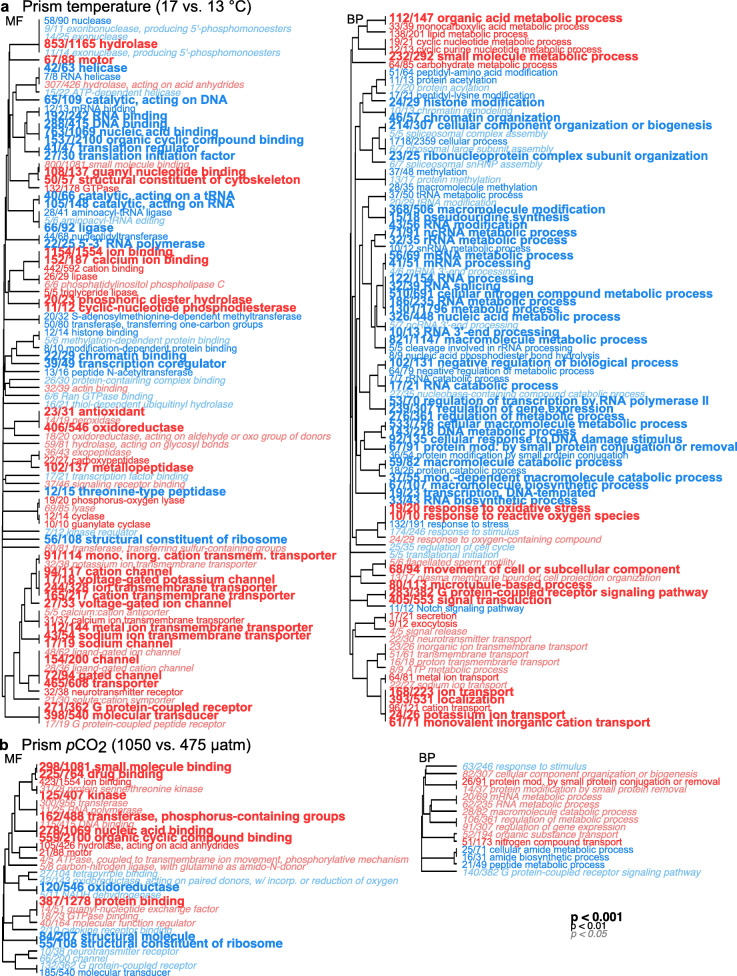
GO results of genes expressed at the prism stage. Analysis determined significant enrichment within GO categories of genes up-regulated (red text) and down-regulated (blue text) due to **a** temperature and **b** *p*CO_2_ treatments in prism embryos. Font sizes of the category names indicate the level of statistical significance as noted in the legend. The fraction preceding each category name is the number of genes with moderated t-statistic absolute values > 1 relative to the total number of genes belonging to the category. GO categories of molecular function (MF) and biological process (BP) are shown

A permutational multivariate ANOVA revealed that at the prism stage, 27.2% of the variance was explained by the temperature treatment (*p* = 0.001) (Fig. 1f). DE analysis showed a total of 3842 genes were up-regulated in embryos raised at 17 °C relative to those raised at 13 °C (Fig. 3c). These up-regulated genes included a proteinase T gene (log_2_ FC = 4.10, adj. *p* < 0.001), a heme-binding protein 2-like gene (log_2_ FC = 3.46, adj. *p* < 0.001), a putative tolloid-like protein 1 gene (log_2_ FC = 3.21, adj. *p* < 0.001), and a heat shock 70 kDa protein 12A-like gene (log_2_ FC = 0.91, adj. *p* < 0.001) (Additional file 3). GO analysis identified GO categories enriched in these up-regulated genes, which included oxidoreductase, response to oxidative stress, ion transmembrane transporter, and ATP metabolic process (Fig. 5a, Additional file 5c). A total of 3434 genes were down-regulated in embryos raised at 17 °C relative to those raised at 13 °C (Fig. 3c), including a toll-like receptor 3 gene (log_2_ FC = − 2.02, adj. *p* < 0.001), a serine/arginine-rich splicing factor 7 gene (log_2_ FC = − 1.19, adj. *p* < 0.001), a heat shock 70 kDa protein cognate 5 gene (log_2_ FC = − 0.21, adj. *p* = 0.021), and a heat shock 70 kDa protein 14 gene (log_2_ FC = − 0.48, *p* < 0.001) (Additional file 3). Enriched GO categories included regulation of gene expression, RNA modification, chromatin organization, cellular response to DNA damage stimulus, and metabolic process (Fig. 5a, Additional file 5c).

The *p*CO_2_ treatment only explained 9.3% of the variance at the prism stage and was not significant (*p* = 0.091) (Fig. 1f). In fact, only 4 genes were up-regulated when comparing the 1050 μatm to the 475 μatm *p*CO_2_ treatment at the prism stage (Fig. 3d), including a hypoxia-inducible factor 1-alpha-like isoform X1 gene (log_2_ FC = 0.84, adj. *p* = 0.005) (Additional file 3). GO categories enriched in up-regulated genes were related to ATPase coupled to transmembrane movement of ions, and regulation of gene expression (Fig. 5b, Additional file 5d). A total of 64 genes were down-regulated in prism embryos raised at 1050 μatm *p*CO_2_ relative to those raised at 475 μatm (Fig. 3d), including a transposon TX1 uncharacterized 149 kDa protein gene (log_2_ FC = − 1.41, adj. *p* = 0.003). Enriched GO terms were related to oxidoreductase and G protein-coupled receptor (Fig. 5b, Additional file 5d). Lastly, the interaction between temperature and *p*CO_2_ factors explained only 7.2% of the variance at the prism stage and was not significant (*p* = 0.362) (Fig. 1f).

## Discussion

In this study, we examined how the gene expression patterns of *M. franciscanus* gastrula and prism embryos varied by the developmental temperature and *p*CO_2_ conditions under which they were raised. We also assessed whether the transcriptomic results aligned with the morphometric and physiological results previously reported in [46]. Although both temperature and *p*CO_2_ can influence rates of sea urchin development [34, 53], any potential differences in developmental timing should not have impacted the results of this study because samples were collected based on developmental progression to the desired embryonic stages as detailed in the Methods, rather than by hours post-fertilization. Overall, we found that while transcriptomic patterns varied by developmental stage, temperature had a dominant effect on changes in gene expression while *p*CO_2_ elicited a more subtle transcriptomic response that was largely limited to the gastrula stage. Experimental conditions impacted genes related to the cellular stress response, transmembrane transport, metabolic processes, and the regulation of gene expression.

In terms of experimental design, embryos were obtained by evenly pooling eggs from five females and fertilizing them with sperm from a single male to produce all full or half siblings. Admittedly, there are caveats to this approach. The results presented here may only be representative of a small subset of the population, or they may be driven by the quality of the particular male selected to fertilize the eggs. Upon including data from our previous study that examined gene expression patterns during *M. franciscanus* early development [48], a PCA showed that, although samples primarily grouped by developmental stage, there is a clear distinction between the embryos of the two studies (Additional file 6). This is likely due to a combination of genetic and environmental differences between the two source populations, as the adult urchins were collected from different sites and during different years. Indeed, in the purple sea urchin *S. purpuratus*, genetic variation has been shown to influence transcriptomic responses to temperature and *p*CO_2_ stress during early development [36, 54]. Given that the data presented here represents a limited selection of the genetic variation that exists in this species, the results should be interpreted with caution. We therefore recommend that additional studies be performed within other *M. franciscanus* populations and with multiple male-female crosses to determine if our results are unique to this study. Nevertheless, this approach was implemented in an effort to limit genetic variability and male-female interactions that may have otherwise confounded the molecular results.

All samples used for RNA extractions were each composed of a pool of 5000 individuals and should thus represent the same mixture of genotypes. Therefore, we do not expect differences in gene expression patterns to be due to genetic variability between embryo cultures, particularly because a low incidence of mortality was observed during the experiment, although it was not directly measured. In the absence of selection, the observed variability in gene expression, body size, and thermotolerance between embryos raised under different experimental treatments reflect plasticity exhibited by *M. franciscanus* during its early development. We discuss this plasticity, and how it may relate to embryo performance under different conditions that *M. franciscanus* are likely to experience in their natural environments currently and in the future under ocean change scenarios.

### Gene expression varied by developmental stage: general patterns

Developmental stage (gastrula or prism embryos) was the primary factor driving differences in gene expression patterns across samples (Fig. 1a and b). In a past study, we raised cultures of *M. franciscanus* embryos in a single laboratory environment that mimicked average, non-stressful conditions in situ (i.e., 15 °C and 425 μatm *p*CO_2_) and documented significant transcriptomic differences between gastrula and prism stages [48]. Therefore, there are many alterations in gene expression between these stages that occur as a result of development and are independent of differences in environmental temperature and/or *p*CO_2_ conditions. This is also evident in Fig. 1a in which gastrula samples do not cluster with prism samples that share the same experimental treatment.

Because comparing gastrula versus prism gene expression patterns was not a goal of this study, no direct differential expression analyses were performed between stages, although gene expression analyses performed independently of stage (i.e., without analyzing gastrula and prism stages separately) are reported in Additional file 2. Nevertheless, embryos at each developmental stage exhibited different transcriptomic responses to temperature and *p*CO_2_ treatments. For instance, many more genes were differentially expressed due to temperature at the prism stage than at the gastrula stage (Fig. 3). Additionally, the *p*CO_2_ treatment explained a significant amount of variance in gene expression in gastrula embryos, but not later at the prism stage (Fig. 1d and f). Similarly, the morphometric response to temperature and *p*CO_2_ treatments varied by stage, in which *p*CO_2_, but not temperature, affected gastrula embryos by reducing body size under elevated *p*CO_2_ conditions (i.e., 1050 μatm) [46]. On the other hand, temperature was the dominant factor at the prism stage, with warmer conditions (17 °C) increasing body size, offsetting the stunting effect of high *p*CO_2_ [46]. The observed patterns between gene expression and body size will be described in greater detail later in the Discussion.

Different life stages are predicted to have different sensitivities to stress [23]. The variability between gastrula and prism stress responses may be explained by a difference in stage-specific vulnerability. During the gastrula stage, the archenteron is formed from invagination of the embryo’s vegetal plate [55], a fundamental process known as gastrulation that is essential for successful development in metazoans [56]. At the prism stage, the embryo differentiates its digestive tract and develops skeletal rods, which are vital structures required for the embryos to eventually become feeding, planktotrophic larvae [57, 58]. Accordingly, differences in responses to environmental conditions between these two stages are likely reflective of the distinct processes undergone by these embryos to ensure their continued developmental progression.

The variability between stages could also be due to the timing and duration of exposure to stress. The effects of a stressor can become increasingly deleterious as the length of exposure continues, and organisms not permitted adequate time to recover may exhibit increasingly poor performance. Furthermore, during development there may be negative carry-over effects that persist into later life stages [59, 60]. Alternatively, organisms may acclimate to stressful conditions over time, and are therefore less adversely affected by a stressor following the initial exposure. For example, in the coral *Acropora hyacinthus*, the immediate transcriptomic response to heat stress was much higher than the transcriptomic response following 20 h of exposure to warmed conditions [61]. Thus, it remains important to acknowledge that organisms may responds differently to various environmental stressors depending on their life history as well as the timing and duration of the exposure.

### Temperature influenced gastrula embryos on a molecular level

Temperature was the dominant factor influencing changes in gene expression at the gastrula stage. Temperature explained 20.3% of the observed variance (*p* = 0.001) with 2049 genes up-regulated and 1955 genes down-regulated in embryos raised under 17 °C relative to those raised under 13 °C (Figs. 1 and 3). Furthermore, PC1, which captured 23.8% of the gene expression variance at the gastrula stage, was significantly correlated to the temperature treatment (Fig. 2a). In general, the observed temperature effects on gene expression at the gastrula stage were approximately akin to those reported for the purple sea urchin *S. purpuratus* [36], whose biogeographical distribution overlaps with that of *M. franciscanus*. Here, DE analysis revealed that gastrula raised in the higher temperature treatment (i.e., 17 °C) expressed genes associated with a cellular stress response. Gastrula embryos of *S. purpuratus* that were raised under an 18 °C temperature treatment exhibited a comparable cellular stress response by up-regulating genes associated with cellular responses to reactive oxygen species and unfolded proteins [36]. We also found that *M. franciscanus* gastrula embryos raised under the warmer treatment exhibited transcriptomic patterns indicative of increased transmembrane transport, while embryos from the colder treatment appeared to decrease metabolic processes. Similarly, *S. purpuratus* increased expression of ion channel, cell-cell signaling, and metabolism genes at warmer temperatures [36]. Lastly, temperature appeared to impact the regulation of gene expression in *M. franciscanus* gastrula embryos, including genes related to epigenetic mechanisms. Temperature also appeared to influence how gene expression was regulated in *S. purpuratus* gastrula embryos with higher temperatures leading to a down-regulation of genes related to transcription and RNA processing [36].

Despite similarities in how temperature influenced gastrula gene expression, unlike in *M. franciscanus*, there was an effect of temperature on *S. purpuratus* morphology. Specifically, *S. purpuratus* gastrula embryos raised at warmer temperatures were significantly smaller in size [36], whereas *M. franciscanus* gastrula embryos did not significantly differ in size as a result of temperature [46]. This could reflect differences in experimental design between the studies (e.g., treatment temperatures, breeding designs, and urchin collection sites), or it could reflect differences that exist at the species level. Unlike *S. purpuratus*, the temperature response of *M. franciscanus* gastrula embryos that occurred at the molecular level was not reflected at the organismal level. We postulate that the transcriptomic differences between gastrula raised at 17 °C and 13 °C served to compensate for direct temperature effects and allowed the embryos to maintain the same size despite the temperature treatments. Below, we explore with greater detail how temperature affected the expression patterns of genes associated with the cellular stress response, transmembrane transport, metabolism, and gene expression regulation in *M. franciscanus* gastrula embryos.

#### Cellular stress response (gastrula temperature)

Response to stress at the cellular level often includes processes to mitigate or remove cell damage [62]. Cell death protein 3, which encodes a protease involved in apoptosis [63], was up-regulated in gastrula embryos raised at 17 °C, indicating that the warmer temperature may have caused stress-induced programmed cell death. DE analysis also provided evidence of DNA damage and repair. This is similar to observations in *Acropora* corals in which heat stress caused an up-regulation of DNA replication and repair genes [61, 64]. Here, GO terms related to DNA metabolic process and DNA recombination were enriched with up-regulated genes under warmer temperature conditions (Fig. 4a). DNA metabolic processes can include both DNA synthesis and degradation for the purposes of replication and repair. Furthermore, DNA recombination in somatic cells has been identified as a critical mechanism for DNA damage repair [65, 66]. Taken together, 17 °C temperature conditions appear to induce stress within the gastrula embryos, which undergo response mechanisms to combat cellular damage.

#### Transmembrane transport (gastrula temperature)

Gastrula embryos raised under warmer temperatures also increased expression of genes related to transmembrane transport, potentially both within and between cells (i.e., cell-cell communication). For instance, genes related to G protein-coupled receptor, cation channel, cell surface receptor signaling pathway, and vesicle-mediated transport were up-regulated in gastrula embryos raised under 17 °C relative to those raised under 13 °C (Fig. 4a). This is also supported by PC loadings, in which genes related to ion binding and cation channel contributed variance to PC1 (Additional file 4a), which was significantly correlated to the temperature treatment (Fig. 2a). Increased transport of materials, particularly ions, across cell membranes may indicate osmoregulation and maintenance of homeostasis. This aligns with reports in juvenile sea urchins of the species *Loxechinus albus*, in which gene expression alterations under elevated temperatures provided evidence of increased active transmembrane transport of sodium and potassium ions [67].

#### Metabolism (gastrula temperature)

In *S. purpuratus*, gastrula embryos raised under warmer temperatures up-regulated metabolic genes [36]. Here, gastrula raised under the lower temperature treatment expressed genes associated with metabolic depression. Specifically, GO terms identified as negative regulation of biological process and negative regulation of metabolic process were significantly enriched by genes down-regulated in gastrula embryos raised under 17 °C relative to those raised under 13 °C (Fig. 4a). In this study, metabolic rates of embryos raised under different treatments were not measured at the gastrula stage, but we may expect that, given the effect of temperature on biochemical reaction kinetics, metabolic rate should increase predictably with temperature [68]. Generally, higher metabolic rates have been recorded at warmer temperatures in marine ectotherms [69–72]. This positive correlation between temperature and metabolism has been observed in *M. franciscanus* at the adult stage [73].

#### Regulation of gene expression (gastrula temperature)

Temperature also had an evident effect on the regulation of gene expression in *M. franciscanus* gastrula embryos. GO terms enriched by genes relatively down-regulated in gastrula embryos raised under 17 °C relative to those raised under 13 °C (i.e., genes were comparatively up-regulated in the colder temperature treatment) were identified as regulation of transcription by RNA polymerase II, translation regulator, and regulation of gene expression (Fig. 4a). Histone binding, histone modification, chromatin remodeling, and chromatin organization genes were also associated with the 13 °C gastrula treatment. Histone and post-translational modifications are examples of epigenetic mechanisms. These, and other epigenetic modifications can act to regulate gene function without altering the DNA sequence, promoting phenotypic plasticity and potentially modulating the response to different environmental conditions [74–76]. Histone variants and modifications may activate or repress transcription processes by altering chromatin structures, impacting the regions of the genome that are available for transcription [77], and have been shown to mediate responses to changing environmental conditions in marine organisms [78–80]. Additional analyses such as ChIP-seq (i.e., chromatin immunoprecipitation sequencing) to locate regions targeted by histone modifications and DNA-binding proteins [81, 82] or ATAC-seq (i.e., assay for transposase-accessible chromatin with high-throughput sequencing) to assess genome-wide chromatin accessibility [83] are required to profile specific histone variants or modifications and their impact on gene expression.

Although evidence of transcription regulation was observed, it is difficult to conclude if this led to an increase or decrease of gene expression, particularly with respect to the functional significance of histone modifications and chromatin remodeling. These mechanisms are much less studied in marine invertebrates than other modifications such as DNA methylation [74, 75], and although our data support that these modifications occurred in response to the environment, additional approaches are required to determine the precise modifications, their locations, and their impact on gene expression. In this study, there were more up-regulated than down-regulated genes in the 17 °C versus the 13 °C gastrula treatment, and the up-regulated genes appeared to have a greater log_2_ FC in expression (Fig. 3a). Nevertheless, future studies pairing comparative epigenetic analyses with transcriptomic approaches are required to elucidate how these various mechanisms influence gene expression in response to different environmental conditions in *M. franciscanus*.

### *p*CO_2_ influenced gene expression of gastrula embryos

The *p*CO_2_ treatment influenced gene expression patterns at the gastrula stage, although to a lesser degree than temperature, and the interaction between the two factors was not significant (Fig. 1d). Gastrula *p*CO_2_ conditions explained 13.2% of the observed variance (*p* = 0.021) with 9 genes up-regulated and 166 genes down-regulated in embryos raised under 1050 μatm *p*CO_2_ relative to those raised under 475 μatm *p*CO_2_ (Figs. 1 and 3). Additionally, PC2, which captured 12.0% of the gene expression variance at the gastrula stage, was significantly correlated to the *p*CO_2_ treatment (Fig. 2a). In this study, we anticipated that the 475 μatm *p*CO_2_ treatment was not stressful, as it represented the average ambient *p*CO_2_ levels *M. franciscanus* regularly experience in their natural habitat [44]. Evidence suggests that calcifying marine organisms such as *M. franciscanus* are sensitive to declines in ocean pH (i.e., increases in *p*CO_2_ levels) [84–86], and while *M. franciscanus* may periodically experience elevated *p*CO_2_ conditions in nature during upwelling events [15, 43, 45, 87], the 1050 μatm *p*CO_2_ treatment was expected to induce a stress response.

While the effect of *p*CO_2_ on gastrula gene expression patterns was less than the effect of temperature, there was a pronounced impact of *p*CO_2_ conditions on gastrula body size. Gastrula raised under elevated *p*CO_2_ conditions (i.e., 1050 μatm) were significantly smaller than those raised under 475 μatm [46]. Therefore, *p*CO_2_ appeared to have a greater influence at the organismal level but elicited a relatively muted transcriptomic response compared to temperature. Below we discuss the expression patterns of genes affected by the gastrula *p*CO_2_ treatment, which included those related to metabolism and ion transport.

#### Metabolism and ion transport (gastrula *p*CO_2_)

Gastrula raised under the lower pCO_2_ treatment (i.e., 475 μatm) expressed genes that significantly enriched GO terms related to several macromolecule biosynthetic processes (Fig. 4b). In contrast, those raised under the elevated *p*CO_2_ treatment (i.e., 1050 μatm) expressed genes that enriched GO terms associated with macromolecule catabolic processes (Fig. 4b). This may, in part, explain the difference in body size that was observed as a result of the *p*CO_2_ conditions. Gastrula in the 475 μatm *p*CO_2_ treatment appeared to construct proteins and other macromolecules to maintain their growth and body size, while gastrula in the 1050 μatm *p*CO_2_ treatment underwent catabolic processes, possibly to obtain the energy required to respond to elevated *p*CO_2_ levels. The *p*CO_2_ stress response may include an increase of ion transport as a means of maintaining acid-base equilibrium given elevated H^+^ concentrations under high *p*CO_2_ conditions. GO terms related to ion binding, active transmembrane transporter, and ATPase coupled to movement of substances were enriched by genes up-regulated in gastrula raised in the 1050 μatm treatment (Fig. 4b). Similarly, increased expression of ion transport genes has been observed in gastrula embryos of *S. purpuratus* exposed to moderately elevated *p*CO_2_ levels (e.g., ~ 800 μatm) [39], although the transcriptomic response of *S. purpuratus* embryos to *p*CO_2_ stress can be influenced by maternal effects [38].

### Temperature was the dominant factor at the prism stage

At the prism stage, temperature accounted for 27.2% of the observed variance (*p* = 0.001) with 3842 genes up-regulated and 3434 genes down-regulated in embryos raised under 17 °C relative to those raised under 13 °C (Figs. 1 and 3). Furthermore, PC1 for the prism stage captured 27.6% of variance in gene expression and was significantly correlated to the temperature treatment (Fig. 2b). Unlike at the gastrula stage, responses to temperature that were measured at the molecular level were also observable at the organismal level. Development at the warmer 17 °C treatment led to an increase in prism body size as well as a modest increase in prism thermotolerance [46]. Indeed, prism body size and thermotolerance variables were also highly correlated to PC1 (Fig. 2b). Therefore, the transcriptomic response to temperature appears to have influenced both growth and resistance to heat stress in *M. franciscanus* prism embryos. Prism embryos raised at 17 °C exhibited increased expression of genes related to the cellular stress response, transmembrane transport, and metabolic processes, while genes related to DNA repair and the regulation of gene expression were associated with the colder temperature treatment (i.e., 13 °C).

#### Cellular stress response (prism temperature)

Environmental stress can lead to the production of reactive oxygen species (ROS), which can cause oxidative stress if ROS production exceeds the organism’s antioxidant or damage repair capacity [88, 89]. Oxidative stress, and the response to resulting cellular damage, due to elevated temperatures have been documented across a wide variety of taxa, including algae [90], plants [91], mollusks [92], and fishes [93, 94]. GO enrichment from the DE analysis identified terms including oxidoreductase, antioxidant, response to oxidative stress, and response to reactive oxygen species that were enriched with genes up-regulated in response to the warmer temperature treatment (Fig. 5a). At the gastrula stage, embryos in the 17 °C treatment expressed genes associated with DNA repair, but there was no evidence of increased macromolecule repair gene expression at the prism stage. This could indicate that while both stages initiated a cellular stress response due to warmer temperatures, there was less cellular damage of nucleic acids incurred by the prism stage.

In general, global change biology research in marine systems has focused on the negative consequences of increasing temperatures associated with ocean warming [95]. However, given the expected rise in variable and extreme weather events, the impact of decreased temperatures is also an important consideration, especially in regions dominated by upwelling. DE analysis indicated that prism embryos in the 13 °C treatment increased expression of genes related to DNA damage and repair. Specifically, identified GO terms included cellular response to DNA damage stimulus, DNA metabolic process, DNA binding, and catalytic, acting on DNA (Fig. 5a). Increased DNA damage as a result of low temperature stress has been recorded in the Pacific white shrimp *Litopenaeus vannamei* [96]. Although 13 °C is within the range of temperatures that *M. franciscanus* experience in the Santa Barbara Channel (SBC) where urchins were collected for this study, it is lower than the annual average of ~ 15 °C [44] and may have generated stress and cellular damage in the prism embryos, leading to the activation of repair mechanisms.

#### Transmembrane transport and metabolism (prism temperature)

Similar to the gastrula stage, warmer temperature conditions caused an increased expression of genes related to transmembrane transport in prism embryos. Specifically, GO terms of ion binding, ion transmembrane transporter, cation channel, and sodium and potassium ion transmembrane transporters were enriched with up-regulated genes (Fig. 5a), which may indicate osmoregulation and the maintenance of homeostasis. Prism embryos in the 17 °C treatment also increased expression of genes related to energetic processes (e.g., ATP metabolic process, organic acid metabolic process, lipid metabolic process, cyclic nucleotide metabolic process, and carbohydrate metabolic process) possibly to generate the energy required to support active transmembrane transport of ions and other materials. The up-regulation of genes related to energy production may have also supported the increased growth of prism embryos under warmer temperatures. In contrast, DE analysis revealed an increase in expression of genes related to the negative regulation of biological and metabolic processes in prism embryos raised at 13 °C. This supports the predicted expectation that organisms exhibit decreased metabolism under colder temperatures [68–72].

#### Regulation of gene expression (prism temperature)

At the prism stage, the colder temperature treatment exhibited an enrichment of genes related to translation regulator, transcription coregulator, regulation of gene expression, and regulation of transcription by RNA polymerase II (Fig. 5a). Additionally, GO terms identified as histone binding, chromatin organization, RNA modification, and methylation-dependent protein binding were enriched in genes down-regulated at 17 °C relative to 13 °C (Fig. 5a). Thus, gene expression in prism embryos raised at 13 °C appeared to be epigenetically regulated by histone and RNA modifications. This differential expression of genes related to gene expression regulation (i.e., down-regulated at the colder temperature relative to the warmer temperature) is similar to the pattern observed at the gastrula stage.

### The prism stage exhibited a limited transcriptomic response to *p*CO_2_

In other studies, echinoderms raised under elevated *p*CO_2_ conditions have exhibited altered expression of genes related to skeletogenic pathways, spicule matrix proteins, cellular stress response, ion regulation and transport, apoptosis, metabolism and ATP production [38, 39, 97–100]. Here, the *p*CO_2_ treatment had a relatively minimal effect on prism gene expression patterns. The *p*CO_2_ treatment explained only 9.3% of the observed variance at this stage and was not significant (*p* = 0.091) (Fig. 1f). This contrasts with observations made at the organism level in which elevated *p*CO_2_ resulted in smaller prism embryos, although this could be offset by the positive effect of temperature, which acted as the dominant factor influencing body size [46]. It is interesting that the transcriptomic response to elevated *p*CO_2_ was more evident in gastrula than in prism embryos particularly because at the prism stage, skeletal rod formation occurs. Although GO terms identified as ion binding and ATPase coupled to transmembrane movement of ions were enriched with genes up-regulated at the elevated *p*CO_2_ level (Fig. 5b), we also expected to observe increased stress associated with prism embryos undergoing calcification processes under lowered pH conditions, but we detected no evidence of this.

It is possible that while there was a clear phenotypic difference in prism embryos raised under high versus low *p*CO_2_ conditions, the transcriptomic changes underlying this difference were much more subtle. Alternatively, the prism stage may simply lack a robust transcriptional response to the 1050 μatm *p*CO_2_ treatment. For instance, the Mediterranean sea urchin *Paracentrotus lividus* exhibits different transcriptomic responses depending on the magnitude of the pH stressor [101]. Decreased pH conditions caused *P. lividus* embryos to increase their expression of calcification genes, but not once the pH dropped below a certain threshold [101]. A similar result was observed in *S. purpuratus* in which embryos raised under a high *p*CO_2_ treatment designed to reflect near-future levels exhibited a muted transcriptomic response relative to those raised under a more moderate *p*CO_2_ treatment designed to reflect present-day low pH conditions [39]. The authors speculated that the transcriptional response required for acclimating to a more extreme *p*CO_2_ level was too metabolically expensive, and the embryos instead opted to conserve energy to ensure short-term survival, perhaps until environmental conditions became more favorable [39]. While a failure of embryos to respond at the transcriptomic level may permit continued successful development under high *p*CO_2_ conditions, there may be important physiological consequences such as the observed reduction in body size [46]. Thus, the lack of a transcriptomic response to high *p*CO_2_ may have important fitness consequences for *M. franciscanus*.

In the green sea urchin *Strongylocentrotus droebachiensis*, a quantitative genetic breeding design implemented by Runcie and colleagues demonstrated that changes in gene expression as a result of differences in pH exposure were minor relative to gene expression differences as a result of parentage [54]. Thus, minimal transcriptomic responses to *p*CO_2_ may be due to the genetic structure of the sea urchins used in this experiment. Furthermore, the environmental exposure history of the adult urchins may have generated non-genetic parental effects (i.e., transgenerational plasticity), which can also generate a limited transcriptomic response to high *p*CO_2_ [38]. While all embryo cultures for this experiment were composed of the same mixture of progeny from a cross between one male and five females, it is possible that the sea urchins collected for this experiment may be from a population with a relatively muted transcriptomic response to high *p*CO_2_ conditions.

It has been proposed that selection and local adaptation act on populations that are regularly exposed to high *p*CO_2_ conditions, such as those that often experience upwelling conditions within the CCS [15], and that these populations may harbor genotypes that are resistant to low pH conditions [40, 98, 102–105]. In *S. purpuratus*, transcriptomic responses to high *p*CO_2_ levels can vary by the frequency in which the sea urchin populations are exposed to upwelling conditions [40]. In particular, urchins from populations frequently exposed to low pH have greater transcriptomic responses to high *p*CO_2_ than those that experience low pH less often [40]. While the site where the adult sea urchins were collected does experience periods of low pH due to upwelling [43, 45], low pH events occur less frequently than at more northern sites within the CCS [15, 106, 107]. Therefore, the urchins used in this study may be comparatively less adapted towards mounting a transcriptomic response to high *p*CO_2_.

### HSP gene expression

Heat shock proteins (HSPs) act as molecular chaperones in the cellular stress response by assisting in protein transport, protein folding and unfolding, stabilization of denatured proteins, and degradation of misfolded proteins [108, 109]. Higher levels of HSPs have been shown to confer increased thermotolerance across a variety of marine taxa [110–113]. Therefore, we may have expected an up-regulation of HSP genes linked with the slight increase in thermotolerance measured at the prism stage [46]. However, there were mixed results regarding differential expression patterns of HSP genes. At both stages, the heat shock 70 kDa protein 12-A like gene was up-regulated in the 17 °C treatment relative to the 13 °C treatment, whereas heat shock 70 kDa protein cognate 5 and heat shock 70 kDa protein 14 were down-regulated. No Hsp70 genes were differentially expressed due to *p*CO_2_, and no Hsp90 genes were differentially expressed due to temperature or *p*CO_2_.

Our results contrast with a study in *S. purpuratus* that found expression of Hsp70 and Hsp90 increased at higher temperatures [36]. However, in other investigations of sea urchin early development, increased Hsp70 expression was generally not observed under moderate warming scenarios. One study found that Hsp70 was not transcriptionally up-regulated in *M. franciscanus* until larvae were exposed to temperatures at or above 20 °C [32]. A study in *S. purpuratus* found induction of Hsp70 only occurred at temperatures above 21 °C [37]. Therefore, the 17 °C treatment may not have been extreme enough to induce a clear differential expression of HSP genes. Furthermore, in the green sea urchin *Psammechinus miliaris*, expression of HSP genes was low during early development relative to expression in adults [114]. The authors suggested that HSP expression was limited during this time [114] because over-expression of HSPs could have negative consequences for successful early development [115]. Therefore, large increases in HSP expression may be restricted during *M. franciscanus* early development.

### Performance under current and future ocean conditions

Moderate ocean warming may be favorable for *M. franciscanus* early development by providing larger body sizes and increased thermotolerance at the prism stage [46]. The warmer temperature treatment could even mitigate the stunting effect of elevated *p*CO_2_ on prism body size [46]. This effect of warmer temperatures, however, may only be beneficial on a short-term basis. Gene expression analyses indicated that embryos raised under 17 °C responded to cellular stress, and while there were no indications of negative impacts at the phenotypic level, there may be trade-offs and consequences to developing under warmer temperatures such as increased incidences of disease [116]. Prolonged heat exposure may eventually become detrimental, and negative carry-over effects can arise at later life stages [59, 60]. Additionally, the observed plasticity at 17 °C may not extend to more severe warming scenarios. For example, a study in adult *M. franciscanus* found that although mortality did not vary between urchins acclimated to 15 °C or 18 °C, mortality was significantly higher at a more extreme temperature of 21 °C [31]. Nevertheless, our study revealed that *M. franciscanus* exhibited a sizeable transcriptomic response to 17 °C at both developmental stages. At the urchin collection site, temperatures of 17 °C are currently recorded during the summer months [44], and in the future, this temperature is likely to be reached more often given unmitigated climate change. More research is required to determine how *M. franciscanus* will be impacted as ocean warming continues, particularly as marine heatwaves increase in frequency [117, 118].

During early development, *M. franciscanus* appear to be more susceptible to rising *p*CO_2_ levels than to rising temperatures. The lack of a large transcriptional response, particularly at the prism stage, paired with a decrease in body size indicates that exposure to elevated *p*CO_2_ is detrimental to developing embryos. Continued ocean acidification is therefore likely to have adverse impacts on future *M. franciscanus* populations, although this could be offset somewhat by the positive effects of simultaneous ocean warming [46, 119–121]. However, during seasonal upwelling events, *M. franciscanus* are exposed to corrosive pH conditions that lack the mitigating effects of warmer temperatures [15, 16]. Spawning that occurs during or immediately prior to an upwelling event will subject developing embryos to high *p*CO_2_ conditions paired with colder temperatures. Values equivalent to the higher *p*CO_2_ treatment (i.e., 1050 μatm *p*CO_2_) have been measured at the urchin collection site during upwelling events [44], so populations of *M. franciscanus* are likely already impacted by elevated *p*CO_2_. Given ocean acidification and the increase in upwelling frequency and intensity that is predicted with continued climate change [122–124], the likelihood of *M. franciscanus* developing under stressful *p*CO_2_ conditions should rise in the future.

## Conclusions

The early developmental stages of *M. franciscanus* exhibited transcriptomic plasticity under different temperature and *p*CO_2_ conditions. The extent of this plasticity, however, varied by developmental stage and by stressor. Although higher temperatures appeared to induce cellular stress, *M. franciscanus* exhibited a robust transcriptional response to temperature. The transcriptomic response to *p*CO_2_ was much more limited and may therefore increase the vulnerability of this species to ocean acidification. We caution against the overgeneralization of these findings because they represent a small portion of the genetic variability that exists in *M. franciscanus*. Future studies that examine different populations and incorporate more genetic diversity will facilitate our understanding of how this species responds to its changing environment.

Accurately predicting how organisms will respond to future ocean conditions is necessary for the implementation of effective conservation and fisheries management strategies. This study provides essential molecular-level insight towards understanding how an ecologically valuable fishery species is affected by climate change. Although we characterized the transcriptomic response of *M. franciscanus* to ecologically relevant temperature and *p*CO_2_ conditions, more work is required to determine how environmental stressors will impact the overall fitness and adaptive potential of natural populations. Detrimental impacts that occur during early development can reduce the quantity or quality of juvenile recruits. Poor recruitment will likely not be immediately evident to the *M. franciscanus* fishery until there is a marked decrease in the number of mature, harvestable adults. This delay in noticeable population declines may cause substantial alterations to coastal macroalgae ecosystems and financial losses to the fishing industry. Studies such as the one presented here are vital for developing proactive and adaptive management strategies to ensure climate-ready fisheries [42, 125].

## Methods

### Animal collection and culturing

Red sea urchins were collected and spawned as described in [46]. Briefly, adults were collected from Ellwood Mesa, Goleta, California, USA (34° 25.065′N, 119° 54.092′W) at 14-m depth via SCUBA on February 21, 2018 under California Scientific Collecting Permit SC-1223 and transported to the Marine Science Institute at the University of California Santa Barbara (UCSB). Spawning was induced by injecting 0.53 M KCl into the coelom through the perioral membrane [20]. Eggs from five individual females and sperm from a single male were collected. A subsample of eggs from each female was fertilized with sperm from the male and high fertilization success was examined for each cross (i.e., visually confirming the formation of fertilization envelopes). These subsamples were only used to verify suitable male-female compatibility and were discarded prior to the experiment. An approximately equal number of eggs from each of the five females were gently pooled together. The pool of eggs was fertilized by slowly adding dilute, activated sperm from the male until approximately 98% fertilization success was reached. Performing crosses with a single male ensured that all cultures were composed of full- or half-sibling embryos. This approach was selected in an effort to limit paternal genetic variability and differences in male-female interactions that could otherwise impact the results of the study.

As described in [46], the newly-fertilized embryos were raised in three replicate culture vessels for each of four different combined temperature and *p*CO_2_ treatments: 1) 17 °C and 1050 μatm *p*CO_2_, 2) 17 °C and 475 μatm *p*CO_2_, 3) 13 °C and 1050 μatm *p*CO_2_, and 4) 13 °C and 475 μatm *p*CO_2_. These treatments represent current conditions measured in the SBC as well as projected future ocean conditions given continuing warming and acidification. It should be noted that the SBC is within a highly dynamic region in which water conditions can vary greatly. For example, the temperature recorded where and when the *M. franciscanus* were collected for this study was 13.3 °C, but throughout the year prior, the temperature at the collection site fluctuated between 10.0–20.5 °C, with an average temperature of 15.1 °C [44]. Static treatments were selected, however, because the early development of *M. franciscanus* occurs rapidly (e.g., < 2.5 days in this study) so unlike their adult counterparts, the embryos are likely to experience relatively stable conditions. The treatment of 13 °C and 475 μatm *p*CO_2_ represents conditions that are regularly measured in the SBC [44]. 13 °C is on the lower end of the range of temperatures observed in this region but is regularly observed during seasonal upwelling events. During upwelling events, combinations of decreased temperatures and elevated *p*CO_2_ levels are common [15, 43, 45, 87]. These upwelling conditions are represented by the combined 13 °C and 1050 μatm *p*CO_2_ treatment. Water conditions in this area also currently reach elevated temperatures of 17 °C on occasion [44], but as ocean warming continues, these conditions are expected to increase in frequency [126]. The combined 17 °C and 1050 μatm *p*CO_2_ treatment represents future conditions as both ocean warming and ocean acidification progress. The temperature, pH, salinity, and total alkalinity were measured in each culture vessel daily as detailed in [46].

### Sample generation and sequencing

The early gastrula stage was designated by the formation of secondary mesenchyme cells and the extension of the archenteron to approximately one-half the embryo’s body length (~ 23.5 h post-fertilization (hpf) at 17 °C and ~ 32.5 hpf at 13 °C). The prism stage was designated by the tripartitioning of the archenteron, the formation of skeletal rods, and the pyramid-like body shape of the embryo (~ 44 hpf at 17 °C and ~ 55.5 hpf at 13 °C). Embryos at both developmental stages were sampled from each culture vessel by gently concentrating the embryos onto a submerged 35-μm mesh filter and transferring them into a 15-mL Falcon tube using a plastic transfer pipet. The concentration of embryos per mL of FSW was calculated by counting three aliquots of embryos so that a coefficient of variance (CV) of less than 10% was reached. At both the gastrula and prism stages, 5000 embryos from each culture vessel were transferred into a 1.5 mL microcentrifuge tube. Embryos were quickly pelleted by centrifugation, excess seawater was removed, and the samples were flash frozen in liquid nitrogen. The samples were stored at − 80 °C until RNA extractions were performed.

Total RNA extractions were performed on samples from each culture vessel at both the gastrula and prism stages for a total of 24 RNA extractions. RNA extractions were performed by adding 500 μL of Trizol® reagent (Invitrogen) to each sample and passing the entire contents through needles of decreasing sizes (i.e., three passes through a 21-gauge needle, a 23-gauge needle, and then a 25-gauge needle) until homogenized. The RNA was isolated using a chloroform addition. The RNA was precipitated in 100% isopropyl alcohol, washed in ice cold 80% ethanol, and resuspended in DEPC-treated water. RNA purity, quantity, and quality were verified using a NanoDrop® ND100, a Qubit® fluorometer, and a TapeStation 2200 system (Agilent Technologies). The RNA samples were submitted to the Genome Center at the University of California, Davis where 24 libraries were generated using poly(A) enrichment. The 24 libraries were pooled and sequenced across two lanes on an Illumina HiSeq 4000 with 100 base-pair (bp) single reads.

### Data processing

Adapter sequence contamination and base pairs with quality scores below 30 were removed from the raw sequence data using Trim Galore! (version 0.4.1) [127]. FastQC (version 0.11.5) [47] was used to verify sequence quality. The trimmed sequence data were mapped onto a developmental transcriptome for *M. franciscanus* [48] and expression values were calculated using RSEM (version 1.3.0) [49] and bowtie2 (version 2.3.2) [128]. The *limma* package [52] in R (version 3.4.4) was used to filter the sequence data to those that had at least 0.5 counts per million mapped reads across at least three of the samples. A scale normalization was applied to the read counts using a trimmed mean of M-values (TMM) normalization method [129]. The counts were converted to log-counts per million (logCPM) using the *cpm* function in *edgeR* [130, 131].

### Principal Component Analysis (PCA)

Principal component analyses (PCAs) were performed on logCPM data to examine distances between the samples using the *PCAtools* package [50] in R. Because gene expression patterns in *M. franciscanus* vary significantly by developmental stage [48] and because comparing gene expression patterns across development was not a goal of the current study, both principal component and differential expression analyses were executed separately for each stage. Using the *eigencorplot* function, PCs were correlated to metadata variables, which included the experiment treatment factors (i.e., temperature and *p*CO_2_). Additional metadata variables were included using data from [46]. Specifically, gastrula and prism body size were included using average length data (mm) for each sample. Thermotolerance, which was only measured for the prism stage, was included using the average LT_50_ value (i.e., the temperature at which 50% mortality occurred) of each treatment. The methods of how body size and thermotolerance values were obtained are described in detail in [46]. *PCAtools* was used to examine the loadings of the PCs with significant correlations to metadata variables. Permutational multivariate ANOVAs were performed using the *adonis* function in the package *vegan* [132] to establish the proportion of variance explained by fixed factors (i.e., developmental stage, temperature treatment, and *p*CO_2_ treatment).

### Differential Expression (DE) analysis

Differential expression (DE) analyses were performed using the limma-trend approach in *limma* by making pairwise comparisons between temperature treatments (17 °C versus 13 °C) and between *p*CO_2_ treatments (1050 μatm versus 475 μatm) for each developmental stage. Moderated *t*-statistics and *p*-values adjusted by the Benjamini and Hochberg’s method were used to control the false discovery rate [133]. Differentially expressed genes (genes relatively down- or up-regulated) were determined for each pairwise comparison of interest (adjusted *p*-value < 0.05). Gene ontology (GO) analyses were performed following the *GO_MWU* package [51] in R. This package uses a rank-based Mann-Whitney U approach to determine whether GO categories are significantly enriched by up- or down-regulated genes. For each comparison, moderated t-test values for all genes were used to test GO enrichment within three categories: molecular function (MF), biological process (BP), and cellular component (CC). The reference transcriptome contains 24,900 contigs linked to GO classifications across 48,990 GO terms [48], however, improved functional annotation may increase the ability to detect biologically relevant information in future *M. franciscanus* RNA-seq analyses.

## Supplementary Information

**Additional file 1.** Summary RNA-seq statistics of all samples.

**Additional file 2. **Differential expression analysis of all samples independent of stage (i.e., gastrula and prism stages not analyzed separately). **a** Principal components that contributed > 80% of the explained variation, PC1-PC7 (columns), were correlated (1 to − 1; *p < 0.05, **p < 0.01, and ***p < 0.001) to metadata variables (rows) of the experiment treatments (i.e., temperature and *p*CO_2_). GO analysis determined significant enrichment within molecular function (MF), biological process (BP), and cellular component (CC) GO categories of genes that contributed variance to **b** PC2, **c** PC3, and **d** PC4. Font sizes of the category names indicate the level of statistical significance as noted in the legend. The fraction preceding each category name is the number of genes with loading absolute values > 0.001 relative to the total number of genes belonging to the category. Genes were differentially expressed (displayed in color) due to **e** the temperature treatment and **f** the *p*CO_2_ treatment. GO analysis determined significant enrichment within GO categories of genes up-regulated (red text) and down-regulated (blue text) due to **g** the temperature treatment and **h** the *p*CO_2_ treatment. The fraction preceding each category name is the number of genes with moderated t-statistic absolute values > 1 relative to the total number of genes belonging to the category.

**Additional file 3. **Lists of genes within each principal component that are significantly correlated to a metadata variable, and lists of genes that are significantly differentially expressed due to temperature or *p*CO_2_ experiment treatments.

**Additional file 4.** GO results of principal component (PC) loadings. The GO analysis determined significant enrichment within molecular function (MF), biological process (BP), and cellular component (CC) GO categories of genes that contributed variance to **a** PC1 and **b** PC2 at the gastrula stage, as well as **c** PC1 and **d** PC2 at the prism stage. Font sizes of the category names indicate the level of statistical significance as noted in the legend. The fraction preceding each category name is the number of genes with loading absolute values > 0.001 relative to the total number of genes belonging to the category.

**Additional file 5. **Additional GO results of differential expression (DE) analyses. The GO analysis determined significant enrichment within the cellular component (CC) category of genes up-regulated (red text) and down-regulated (blue text) due to **a** the temperature treatment in gastrula embryos, **b** the *p*CO_2_ treatment in gastrula embryos, **c** the temperature treatment in prism embryos, and **d** the *p*CO_2_ treatment in prism embryos. Font sizes of the category names indicate the level of statistical significance as noted in the legend. The fraction preceding each category name is the number of genes with moderated t-statistic absolute values > 1 relative to the total number of genes belonging to the category.

**Additional file 6. **Principal component analysis (PCA) plot of all samples as well as samples from a previous study that examined gene expression patterns during the early development of *M. franciscanus* [48].

## Data Availability

The datasets generated and/or analyzed during the current study are available in the NCBI Short Read Archive under Bioproject accession number PRJNA637102. Additional R data and analysis scripts are available at a GitHub repository (https://github.com/julietmwong27/Mfranciscanus_RNAseq_Ranalysis).

## Abbreviations

*p*CO_2_: Partial pressure of carbon dioxide
RNA-seq: RNA sequencing
PCA: Principal component analysis
PC: Principal component
DE: Differential expression
GO: Gene ontology
FC: Fold change
MF: Molecular function
BP: Biological process
CC: Cellular component
mm: Millimeters
ROS: Reactive oxygen species
ChIP-seq: Chromatin immunoprecipitation sequencing
ATAC-seq: Assay for transposase-accessible chromatin with high-throughput sequencing
rRNA: Ribosomal RNA
HSP: Heat shock protein
CCS: California Current System
UCSB: University of California Santa Barbara
SBC: Santa Barbara Channel
hpf: Hours post-fertilization
CV: Coefficient of variance
bp: Base-pair
TMM: Trimmed mean of M-values
