# Supplementary figures and images for: Gene expression patterns of red sea urchins (Mesocentrotus franciscanus) exposed to different combinations of temperature and pCO2 during early development
Source: BMC Genomics. 2021 Jan 7;22:32. doi: 10.1186/s12864-020-07327-x (PMC7792118; doi:10.1186/s12864-020-07327-x)

**a**

Correlates

Temp (°C)  
  
pCO<sub>2</sub> (μatm)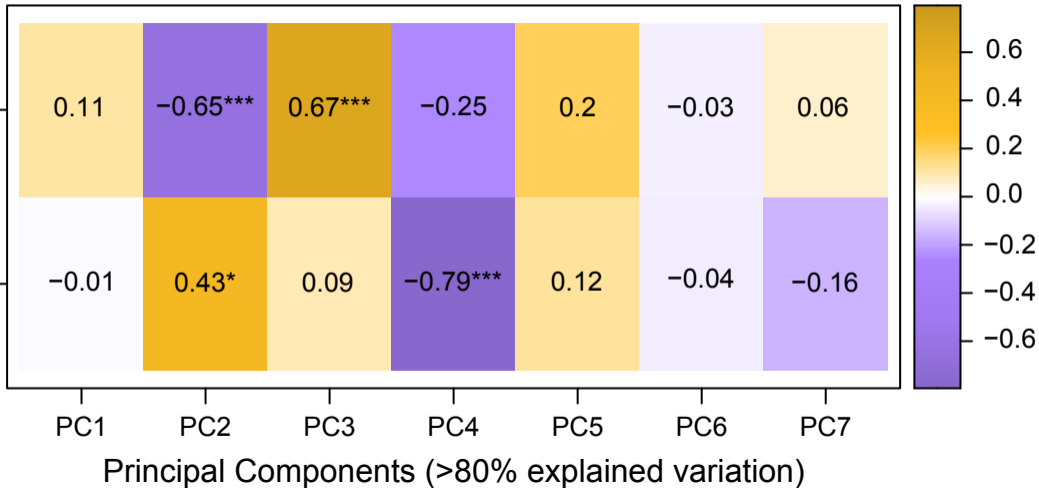

**b** PC2 (3.8%)

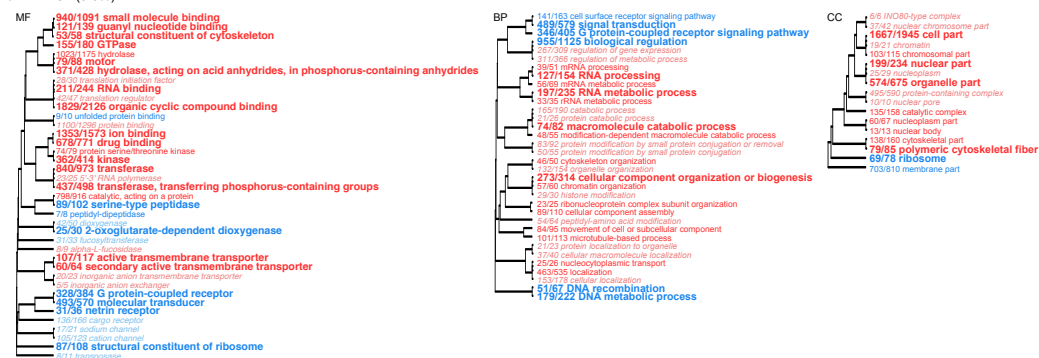

**c** PC3 (3.2%)

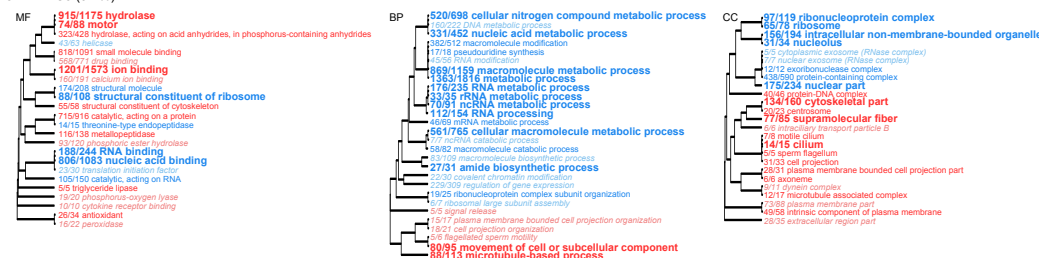

**d** PC4 (2.2%)

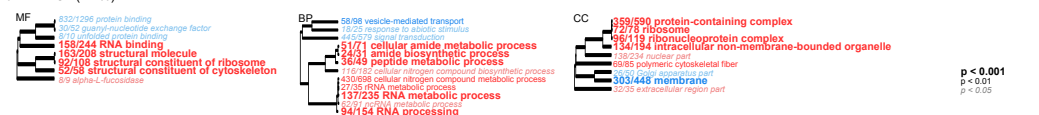

p < 0.001  
p < 0.01  
p < 0.05

**e** Temperature (17 vs. 13 °C)

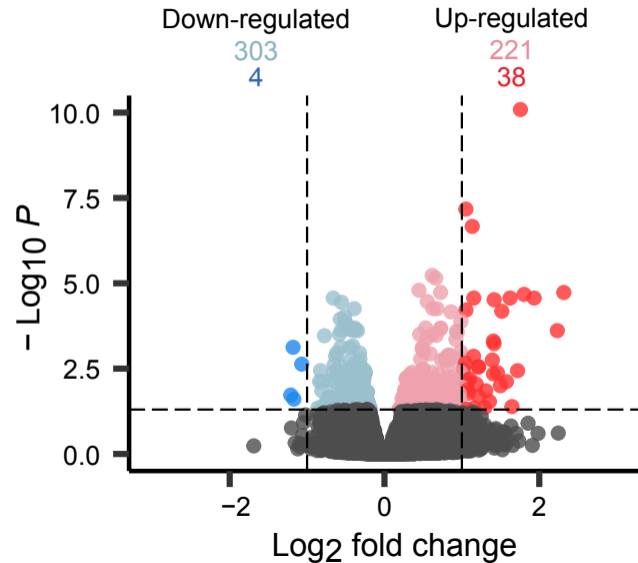

**f**  $p\text{CO}_2$  (1050 vs. 475  $\mu\text{atm}$ )

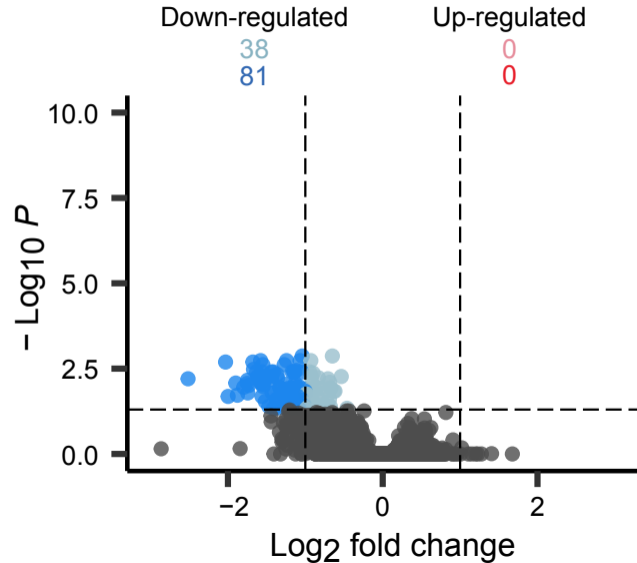

## a

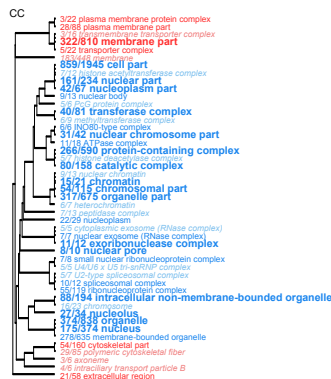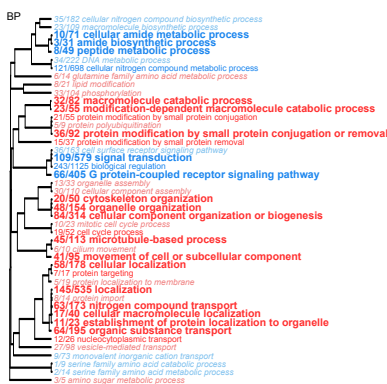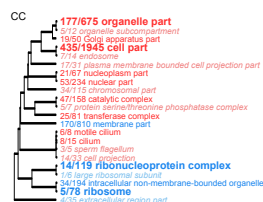

**p < 0.001**  
p < 0.01  
p < 0.05

Supplement: Supplementary file 2 — Additional file 2. Differential expression analysis of all samples independent of stage (i.e., gastrula and prism stages not analyzed separately). a Principal components that contributed > 80% of the explained variation, PC1-PC7 (columns), were correlated (1 to − 1; *p < 0.05, **p < 0.01, and ***p < 0.001) to metadata variables (rows) of the experiment treatments (i.e., temperature and pCO2). GO analysis determined significant enrichment within molecular function (MF), biological process (BP), and cellular component (CC) GO categories of genes that contributed variance to b PC2, c PC3, and d PC4. Font sizes of the category names indicate the level of statistical significance as noted in the legend. The fraction preceding each category name is the number of genes with loading absolute values > 0.001 relative to the total number of genes belonging to the category. Genes were differentially expressed (displayed in color) due to e the temperature treatment and f the pCO2 treatment. GO analysis determined significant enrichment within GO categories of genes up-regulated (red text) and down-regulated (blue text) due to g the temperature treatment and h the pCO2 treatment. The fraction preceding each category name is the number of genes with moderated t-statistic absolute values > 1 relative to the total number of genes belonging to the category. [file 12864_2020_7327_MOESM2_ESM.pdf]

**a** Gastrula PC1 (23.8%)

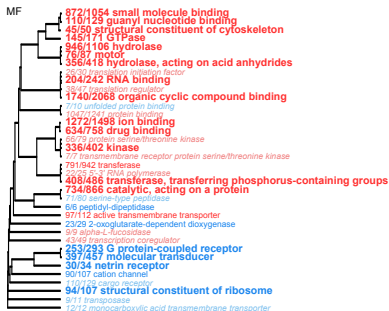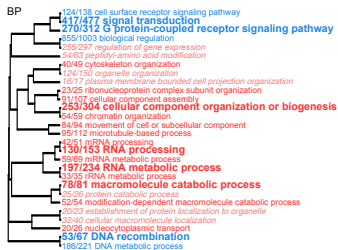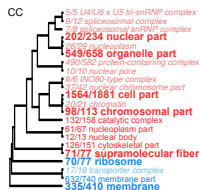

**b** Gastrula PC2 (12.0%)

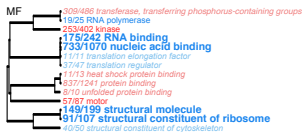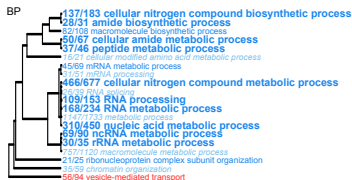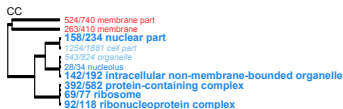

p < 0.001  
p < 0.01  
p < 0.05

Supplement: Supplementary file 4 — Additional file 4. GO results of principal component (PC) loadings. The GO analysis determined significant enrichment within molecular function (MF), biological process (BP), and cellular component (CC) GO categories of genes that contributed variance to a PC1 and b PC2 at the gastrula stage, as well as c PC1 and d PC2 at the prism stage. Font sizes of the category names indicate the level of statistical significance as noted in the legend. The fraction preceding each category name is the number of genes with loading absolute values > 0.001 relative to the total number of genes belonging to the category. [file 12864_2020_7327_MOESM4_ESM.pdf]
