# Supplementary material for: Gene expression patterns of red sea urchins (Mesocentrotus franciscanus) exposed to different combinations of temperature and pCO2 during early development
Source: BMC Genomics. 2021 Jan 7;22:32. doi: 10.1186/s12864-020-07327-x (PMC7792118; doi:10.1186/s12864-020-07327-x)
Supplement: Supplementary file 5 — Additional file 5. Additional GO results of differential expression (DE) analyses. The GO analysis determined significant enrichment within the cellular component (CC) category of genes up-regulated (red text) and down-regulated (blue text) due to a the temperature treatment in gastrula embryos, b the pCO2 treatment in gastrula embryos, c the temperature treatment in prism embryos, and d the pCO2 treatment in prism embryos. Font sizes of the category names indicate the level of statistical significance as noted in the legend. The fraction preceding each category name is the number of genes with moderated t-statistic absolute values > 1 relative to the total number of genes belonging to the category. [file 12864_2020_7327_MOESM5_ESM.pdf]

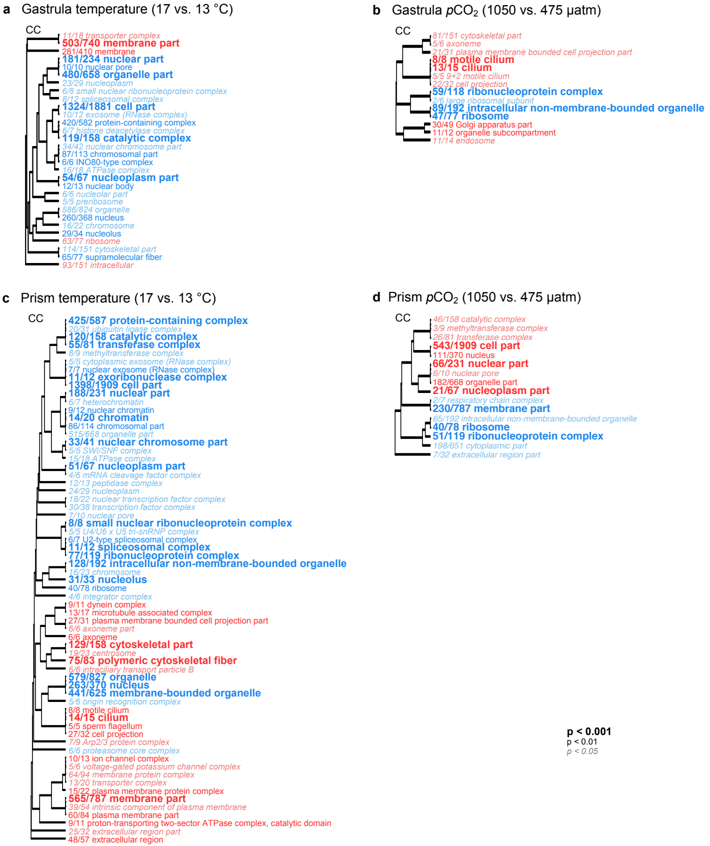
a Gastrula temperature (17 vs. 13 °C)
CC
11/18 transporter complex
503/740 membrane part
281/410 membrane
181/234 nuclear part
10/10 nuclear pore
480/658 organelle part
23/29 nucleoplasm
6/8 small nuclear ribonucleoprotein complex
8/12 spliceosomal complex
1324/1881 cell part
10/12 exosome (RNase complex)
420/582 protein-containing complex
6/7 histone deacetylase complex
119/158 catalytic complex
34/42 nuclear chromosome part
87/113 chromosomal part
6/6 INO80-type complex
16/18 ATPase complex
54/67 nucleoplasm part
12/13 nuclear body
6/6 nucleolar part
5/5 preribosome
586/824 organelle
260/368 nucleus
16/22 chromosome
29/34 nucleolus
63/77 ribosome
114/151 cytoskeletal part
65/77 supramolecular fiber
93/151 intracellular
b Gastrula pCO2 (1050 vs. 475 µatm)
CC
81/151 cytoskeletal part
5/6 axoneme
21/31 plasma membrane bounded cell projection part
8/8 motile cilium
13/15 cilium
5/5 9+2 motile cilium
22/32 cell projection
59/118 ribonucleoprotein complex
2/6 large ribosomal subunit
89/192 intracellular non-membrane-bounded organelle
47/77 ribosome
30/49 Golgi apparatus part
11/12 organelle subcompartment
11/14 endosome
c Prism temperature (17 vs. 13 °C)
CC
425/587 protein-containing complex
20/31 ubiquitin ligase complex
120/158 catalytic complex
55/81 transferase complex
8/9 methyltransferase complex
5/5 cytoplasmic exosome (RNase complex)
7/7 nuclear exosome (RNase complex)
11/12 exoribonuclease complex
1398/1909 cell part
188/231 nuclear part
6/7 heterochromatin
9/12 nuclear chromatin
14/20 chromatin
86/114 chromosomal part
515/668 organelle part
33/41 nuclear chromosome part
5/5 SWI/SNF complex
15/18 ATPase complex
51/67 nucleoplasm part
4/6 mRNA cleavage factor complex
12/13 peptidase complex
24/29 nucleoplasm
18/22 nuclear transcription factor complex
30/38 transcription factor complex
7/10 nuclear pore
8/8 small nuclear ribonucleoprotein complex
5/5 U4/U6 x U5 tri-snRNP complex
6/7 U2-type spliceosomal complex
11/12 spliceosomal complex
77/119 ribonucleoprotein complex
128/192 intracellular non-membrane-bounded organelle
16/23 chromosome
31/33 nucleolus
40/78 ribosome
4/6 integrator complex
9/11 dynein complex
13/17 microtubule associated complex
27/31 plasma membrane bounded cell projection part
6/6 axoneme part
6/6 axoneme
129/158 cytoskeletal part
19/23 centrosome
75/83 polymeric cytoskeletal fiber
6/6 intraciliary transport particle B
579/827 organelle
263/370 nucleus
441/625 membrane-bounded organelle
5/6 origin recognition complex
8/8 motile cilium
14/15 cilium
5/5 sperm flagellum
27/32 cell projection
7/9 Arp2/3 protein complex
6/6 proteasome core complex
10/13 ion channel complex
5/6 voltage-gated potassium channel complex
64/94 membrane protein complex
13/20 transporter complex
15/22 plasma membrane protein complex
565/787 membrane part
39/54 intrinsic component of plasma membrane
60/84 plasma membrane part
9/11 proton-transporting two-sector ATPase complex, catalytic domain
25/32 extracellular region part
48/57 extracellular region
d Prism pCO2 (1050 vs. 475 µatm)
CC
46/158 catalytic complex
3/9 methyltransferase complex
26/81 transferase complex
543/1909 cell part
111/370 nucleus
66/231 nuclear part
6/10 nuclear pore
182/668 organelle part
21/67 nucleoplasm part
2/7 respiratory chain complex
230/787 membrane part
65/192 intracellular non-membrane-bounded organelle
40/78 ribosome
51/119 ribonucleoprotein complex
198/651 cytoplasmic part
7/32 extracellular region part
p < 0.001
p < 0.01
p < 0.05
