# Supplementary material for: Gene expression patterns of red sea urchins (Mesocentrotus franciscanus) exposed to different combinations of temperature and pCO2 during early development
Source: BMC Genomics. 2021 Jan 7;22:32. doi: 10.1186/s12864-020-07327-x (PMC7792118; doi:10.1186/s12864-020-07327-x)
Supplement: Supplementary file 6 — Additional file 6. Principal component analysis (PCA) plot of all samples as well as samples from a previous study that examined gene expression patterns during the early development of M. franciscanus [48]. [file 12864_2020_7327_MOESM6_ESM.pdf]

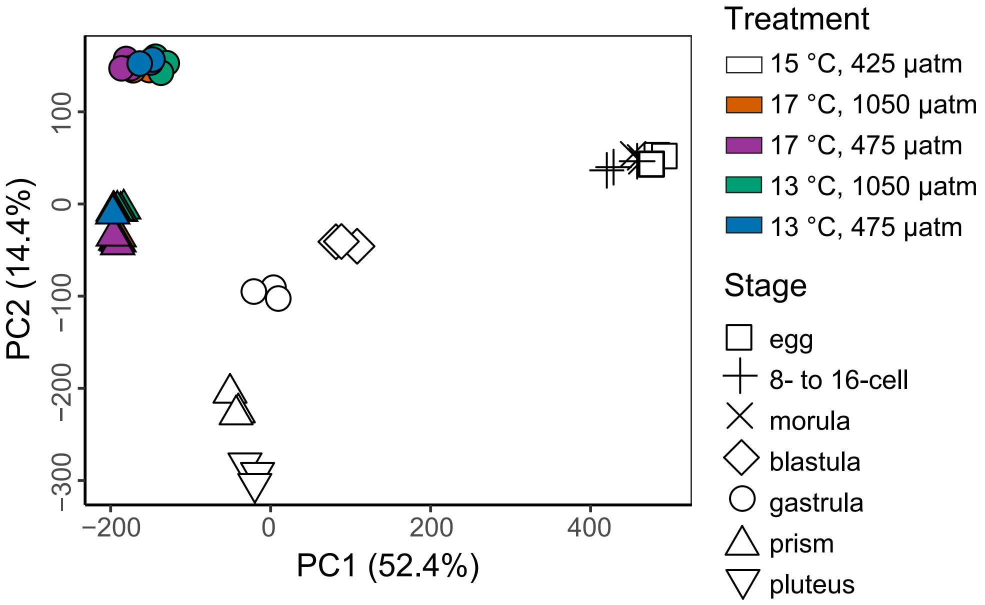

Treatment
15 °C, 425 µatm
17 °C, 1050 µatm
17 °C, 475 µatm
13 °C, 1050 µatm
13 °C, 475 µatm
Stage
egg
8- to 16-cell
morula
blastula
gastrula
prism
pluteus
PC2 (14.4%)
100
0
−100
−200
−300
−200
0
200
400
PC1 (52.4%)
